# Diagnosis and treatment of congenital nasal dermoid and sinus cysts in 11 infants: A consort compliant study

**DOI:** 10.1097/MD.0000000000019435

**Published:** 2020-05-22

**Authors:** Kun Ni, Xiaoyan Li, Limin Zhao, Jiali Wu, Xiaojun Liu, Haibo Shi

**Affiliations:** a Department of Otolaryngology-Head and Neck Surgery, Shanghai Children's Hospital, Shanghai Jiao Tong University; b Department of Otolaryngology-Head and Neck Surgery, Shanghai Sixth People's Hospital, Shanghai Jiao Tong University, Shanghai Key Laboratory of Sleep Disordered Breathing, Shanghai, China.

**Keywords:** congenital midline nasal dermoids, infant, nasal endoscopy, vertical incision

## Abstract

There have been few studies on congenital nasal dermoid and sinus cysts (NDSCs) in infants.

This study was performed to obtain clinical data for the diagnosis and treatment of NDSCs in infants.

We performed a retrospective analysis of 11 infants admitted with NDSCs between 2014 and 2019. Patient demographics, lesion site, preoperative radiological findings, surgical technique, intraoperative findings, and postoperative sequelae were analyzed.

In total, 11 infants (average age, 19 months; lowest age, 10 months) were included in this study. All patients presented with a nasal root mass, 2 patients also had nasal tip fistula, and only 1 case had a history of preoperative infection. Preoperative enhanced computed tomography (CT) examination showed nasal surface lesion (type I) in 3, nasal intraosseous (type II) in 5, intracranial epidural (type III) in 2, and intracranial dural (type IV) in 1 patient. The main surgical methods included direct resection with a vertical midline incision (9 patients), vertical incision + transnasal endoscopic resection + skull base repair (1 patient), and transverse incision of the lower margin of the left eyebrow (1 patient). All wounds healed well without serious complications.

Using the 4-type classification method in combination with the preoperative CT findings to analyze the extent of NDSC in infants is helpful for formulating the surgical plan. Using vertical incision approach alone or combined with nasal endoscopy for minimally invasive surgery can meet the needs of complete resection and reconstruction.

Our results provide clinical data that can help establish standardized criteria for the diagnosis and treatment of NDSCs in infants.

## 1 Introduction

Congenital nasal dermoid and sinus cysts (NDSCs) are rare congenital malformations, not commonly observed in clinics. They are the most common type of congenital nasal midline lesions.^[1]^ Other types include nasal meningoencephaloceles and gliomas. The incidence of dermoid cysts and fistulas in the midline of the nose is 1/20,000 to 1/40,000.^[1]^ NDSCs constitute approximately 11% of dermoid cysts in the head and neck, 1% of dermoid cysts in the whole body,^[2]^ and 61% of median lesions in children.^[3]^

The main characteristics of NDSCs are as follows: the fistula orifice is located on the median line of the nose, sometimes the fistula orifice could be located on the median line of the face between the eyebrow and nasal columella, and sometimes there is a second fistula in the inner canthus. the fistula presents as a needle-like orifice with white cheese-like substance or fine hair discharged after extrusion; the cyst presents as an elastic round mass at the median line of the nose; both dermoids and cysts could occur repeatedly following infection; severe patients can even be complicated by meningitis, cellulitis, osteomyelitis, cerebrospinal fluid leakage, frontal abscess, and dead bone formation; inward growth of the lesions can reach the nasal bone or nasal septum cartilage; intracranial invasion is observed in 20% of patients, whereas few patients can present with widening of the orbital space.^[4]^ Local lesions in the midline of the nose can be classified as fistulas, cysts, or mixed type. Diagnosis is made based on the symptoms and signs and computed tomography (CT)/magnetic resonance imaging (MRI) examination. The differential diagnoses include nasal meningoencephalocele and glioma. Some researchers^[5]^ classify the NDSCs as the nasal surface type, nasal intraosseous type (with invasion of the cartilage and bone), intracranial epidural type, and intracranial dural type according to the extent of the lesions. The extent of the lesions is judged mainly by preoperative CT images and intraoperative exploration.

NDSCs are usually treated by surgery. Surgical methods for removal of the dermoid and cyst should meet the following requirements: optimal access for complete removal of the cyst, fistula, and diseased bone tissue; repair of the skull base to stop cerebrospinal fluid leakage; promote nasal reconstruction; and postoperative cosmesis. Head and neck surgeons should consider various surgical methods to resolve these problems. Since NDSCs were formally identified in 1982, many surgical methods have been proposed, ranging from the initial invasive nasal lateral incision, double coronal flap incision, and frontal craniotomy to the trauma-reducing eyebrow incision, small frontal window craniotomy, and progressive osteotomy, and finally, to the minimally invasive vertical midline incision, transnasal endoscopic rhinoplasty, and nasal endoscopic intracranial lesion incision plus anterior skull base repair.

In this study, we collected the data of patients admitted to our department with the diagnosis of congenital NDSC from 2014 to 2019. As per the latest classification method, NDSCs were classified as 4 types. Type I was nasal surface type, type II was nasal intraosseous type (with invasion of the cartilage and bone), type III was intracranial epidural type, and type IV was intracranial dural type. This study analyzed the case characteristics, surgical methods, and prognosis with the aim of providing clinical data to establish standardized criteria for the diagnosis and treatment of NDSC.

## 2 Methods

### 2.1 Patient characteristics

Eleven infants and young children diagnosed with NDSC were treated in our hospital from 2014 to 2019. Informed consent was obtained from the patients’ parents to use patient information in this study. The research program was approved by the Ethics Committee of Shanghai Children's Hospital (No. 2019R094_2019-01-3). All patients presented with a nasal root mass, and 2 patients included a nasal tip fistula. The main symptoms were observed in the midline or the left or right sides as a round nasal mass immediately after birth.

### 2.2 Diagnostic criteria

The following diagnostic criteria were used: rounded mass or pinpoint orifice on the midline of the nose found immediately after birth, and white cheese-like substance or fine hair discharged after extrusion, or both; a history of local recurrent infection; CT and/or MRI showing a soft tissue mass at any position on the midline of the nose. Patients of meningoencephalitis, teratomas, and lipomas were excluded.

### 2.3 Supplementary examination

B-mode ultrasonography showed the local nasal tumors and confirmed a hypoechoic soft tissue shadow. Further enhanced CT examination was performed to determine the extent of the mass, and to see whether there was destruction of the nasal and frontal bones, whether it was connected with the skull base, and whether there was intracranial invasion.

## 3 Results

Eleven infants were included in this study, average age 19 months, range 9 to 27 months, and 9 men and 2 women. Four patients presented with slow-growing mass; 10 had no history of infection; and 1 patient had local redness, swelling, and abscess after surgery. All patients underwent surgical treatment. Nine patients underwent vertical incision on the nose and face, 1 underwent transverse incision on the lower margin of the left eyebrow according to the location of the mass, 1 underwent double incision for recurrence of infection and fistula, and 2 underwent vertical incisions on the focus of infection. The incision pattern depended on the need for complete excision and aesthetic principles. After the operation, 5-0 cosmetic suture was used intradermally, and the wound was fixed with bioglue. In 1 patient, where the lesion had invaded the intracranial space, endoscopy was used to enter the skin incision in the nasal cavity to help with complete excision of the lesion. Cerebrospinal fluid leakage occurred after excision of the lesion. Three-layer repair of the anterior skull base defect was performed with myofascial suturing, gelatin sponge, and bioglue.

Based on the latest classification method and the results of CT images, 11 children were preoperatively classified as NDSC. In 3 patients, the lesion was confined to the subcutaneous part of the middle nasal line without bone compression or destruction, and these were classified as type I. Five patients were classified as type II with obvious nasal bone destruction and local depression. In 2 patients (patients 9 and 10), the lesion was connected with the anterior skull base without obvious bone defect, there was no intracranial invasion, and these were classified as type III. Only 1 (patient 11) patient was classified as type IV, with intracranial involvement and local bone defect in the anterior skull base (Table 1).

**Table 1:**
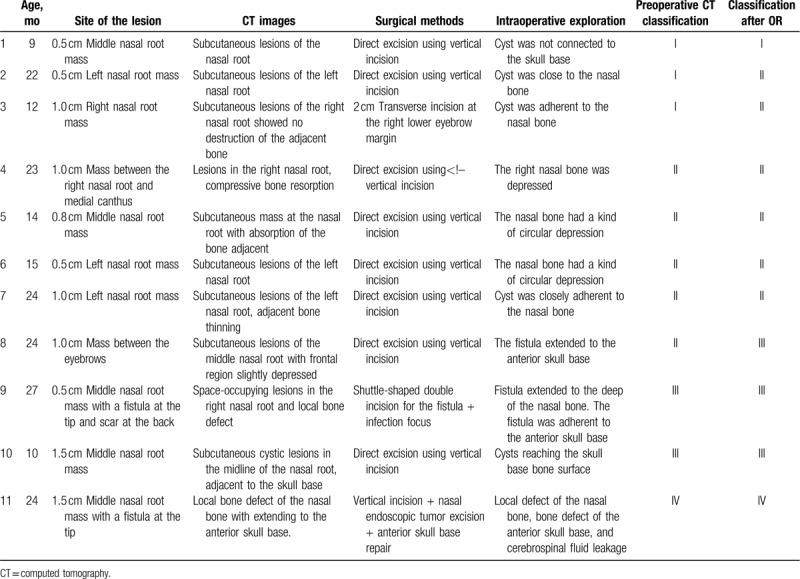
Clinical data of cases.

During the operation, in 1 patient, the lesion was found to be confined to the subcutaneous part of the midline of the nose, without adhesion to the nasal bone and bone compression or destruction. In the other 3 patients that were preoperatively diagnosed as type I by CT, intraoperative exploration showed that the mass was closely related to the nasal bone. One fistula ended in the nasal bone, and 2 had adhesions to the nasal bone. These 3 patients were corrected to type II postoperatively. Five patients diagnosed as type II preoperatively were found to have obvious nasal bone destruction or local depression and bone absorption during the operation. In 1 patient, the lesion was traced to the anterior skull base, and was adherent to the skull base. Postoperatively, this case was classified as type III. In a patient with type III lesion, the fistula was observed to extend from the subcutaneous area to the deep surface of the nasal bone, with obvious adherence to the anterior skull base above. No obvious defect was observed in the skull base in 1 patient with type IV lesion. Local defect of the left nasal bone, cyst connected with the dura mater of the anterior skull base, bone defect of the anterior skull base, and cerebrospinal fluid leakage were found after cyst removal. Postoperative classification of the lesions included 1 patient of type I, 7 of type II, 3 of type III, and 1 of type IV. Thus, there were some inconsistencies between preoperative CT classification and classification after intraoperative exploration; There were 9 patients of coincidence between preoperative CT classification and intraoperative exploration (81.8%) and 2 patients of inconsistency (18.2%). Classification by CT could, however, provide a reference for accurate surgical design. All children had different degrees of postoperative wound swelling, which subsided after 3 to 5 days. Fever developed in 5 patients postoperatively. One patient with type IV had high fever postoperatively, with body temperatures of 39°C that lasted for 3 days, and 38°C to 38.5°C that lasted for 3 days; subsequently, the patient improved. There was no cerebrospinal fluid rhinorrhea, mental distress, crying, vomiting, or other symptoms postoperatively. One month postoperatively, all children had good wound healing, no obvious local deformity.

## 4 Discussion

In this study, we analyzed the case characteristics, surgical methods, and prognosis of infants and children with NDSC. Our results provide clinical data that can help establish standardized criteria for the diagnosis and treatment of NDSC.

There are 2 temporary gaps during the development of the nasofrontal region. One is the small fontanel, the nasofrontal fontanel, which is located between the nasofrontal bones on both sides. The second is the anterior nasal space, the posterior nasal sac, and the anterior nasal bone and fontanel. At the end of the second month of embryonic development, the meningeal diverticulum extends forward and downward through the anterior nasal space. During normal development, the frontal and nasal processes grow forward and downward to surround and protrude through the meninges, forming a tubular blind hole. The prominent meninges eventually degenerate into fibrous structures and fill in the eliminated pores. Dermoid cyst, meningoencephalocele, and glioma of the nose all occur around normal points of development. Brunner and Harned,^[6]^ Sessions,^[7]^ and others elaborated on the pathogenesis of dermoid cysts in the nose. They believed that dermoid cysts in the back of the nose, some nasal gliomas, and meningoencephaloceles originated from incomplete degeneration of the meningeal protrusion that crosses the anterior nasal space for a short time. If the meningeal protrusion occurred close to the skin, small pits would appear in the outer nose, that is, the outer foramen of the dermoid cyst in the back of the nose. The medial fistula can eventually be located in any part of the meningeal protrusion path. Dermoid or epithelioid tumors enlarge due to epithelial debris in the inner wall of the mass. The mass can extend upward through the blind foramen to the anterior cranial fossa, backward to the lower sieve plate, and forward to the fontanel.

**Figure 1:**
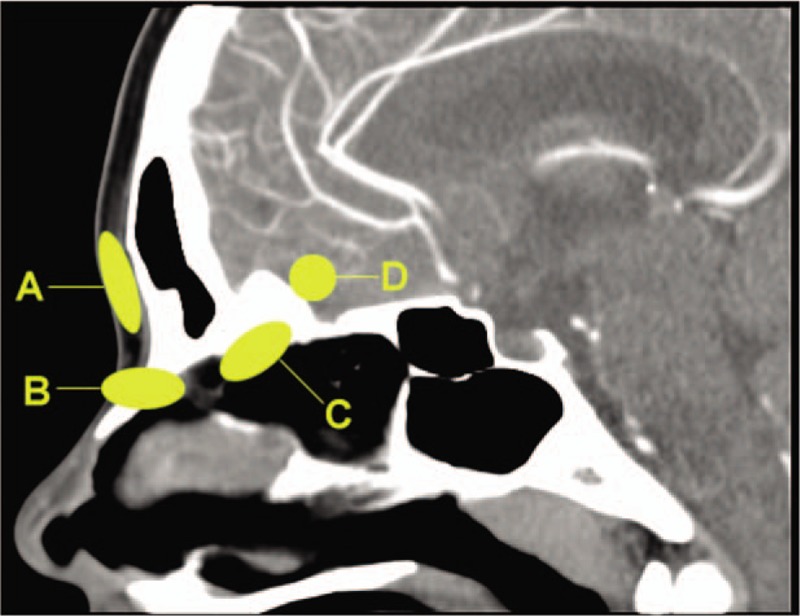
Illustration of the 4 parts of the lesion in computed tomography images. A, A type I lesion. B, A type II lesion. C, A type III lesion. D, A type IV lesion.

**Figure 2:**
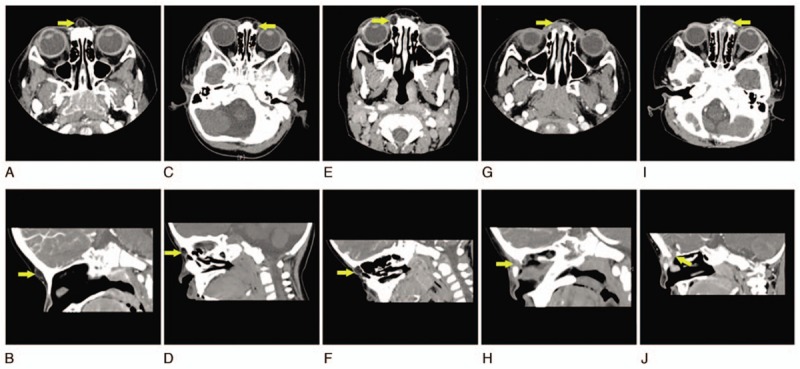
Computed tomography (CT) findings of each type among patients in this study. A and B, A type I lesion on horizontal and sagittal CT in the same patient. C and D, A type II lesion in 1 patient. E and F, A type II lesion in another patient. G and H, A type III lesion. I and J, A type IV lesion. The relationships of the lesion, nasal bone, and anterior skull base can be clearly observed in the images.

Figure 1 shows an illustration of the 4 parts of the lesion. Location A shows the cyst located subcutaneously, with or without fistula orifice in the external nose, and not invading the nasal or frontal bones; this was a type I lesion. Location B shows the lesion involving the nasal septal cartilage or nasal bone, but not reaching the intracranial area; this was a type II lesion. Locations C and D show the lesion connected with the intracranial area, with a possible bone defect of the skull base. Lesions in location D are often combined with intracranial mass or skull base bone defects. We defined these as types III and IV, respectively. Thus, our study included patients ranging from type I to type IV. The CT findings of each type are shown in Figure 2. In type I, the lesions were confined to the subcutaneous part of the dorsum of the nose (Fig. 2A, B). In type II, the masses were connected to the nasal bone, and there were some bone defects (Fig. 2C–F). In type III, the masses destroyed the nasal bone and were associated with the anterior skull base, but no intracranial mass was observed (Fig. 2G–H). In type IV, the masses invaded the nasal bone and extended to the skull; internally, there were skull base bone defects (Fig. 2I–J). In some patients, there was no invasion of the nasal bone on CT, but intraoperative exploration revealed that the fistula penetrated into the nasal bone. Furthermore, in some patients, the lesions seemed unrelated to the skull base on preoperative CT, but intraoperative exploration revealed that extensions of the lesions were closely connected with the anterior skull base. This suggests that CT examination can be used for preliminary classification and help with surgical planning, but determination of the final extent of the lesion requires careful intraoperative investigation. Our classification method is consistent with that proposed by Hartley et al.^[5]^ Congenital midline nasal deformity is a rare disease that may be associated with intracranial extension and an abnormal anterior skull base. Safe surgical treatment of these lesions depends on accurate preoperative imaging, which can help establish the diagnosis, guide surgical planning, and aid communication regarding the diagnosis and surgical methods; thus, it can help to optimize family counseling.

Treatment of NDSCs involves resection of the lesion as soon as possible after diagnosis. Some clinicians recommend that the best age for resection is at 2 years of age.^[8]^ Others believe that selective surgery with close follow-up is optimal for patients without an abnormal external nose, no obvious growth of the lesions, and no history of infection. However, the presence of space-occupying lesions may affect the development of local structures with age. As the volume of space-occupying lesions increases gradually, compression of the bone and repeated infections may occur, thus increasing the difficulty of surgery and postoperative repair. Therefore, we recommend early resection. If infection has occurred, it is recommended that the infection be controlled first, followed by surgery as soon as possible. It has been reported that cysts with a recessive cranial fissure^[9]^ and NDSCs extending into the intracranial space and causing infectious abscess^[10]^ could have long-term adverse consequences. In this study, the average age of presentation was 19 months, with patients occurring as early as at 10 months. There were no serious complications and the prognosis was good. The results of the operation in young children indicate that age should not be a restricting factor for surgery.

NDSC treatment has evolved over the past 40 years, and there have been many reports regarding surgical methods for intracranial extension of NDSCs. The recommended surgical method for type I NDSC is direct resection. The surgery can be performed using 2 types of incisions, horizontal and vertical. The principle of the incision is to fully expose the lesion and attain optimum cosmesis. Some clinicians have used transverse nasal incisions and cosmetic sutures, and achieved satisfactory results.^[11]^ Some clinicians also believe that the vertical midline incision combined with modifications for skin excision can provide sufficient exposure for complete excision and local reconstruction of nose and is a satisfactory cosmetic method for nasofrontal masses.^[12]^ Type II NDSC is often accompanied by destruction of the nasal structures. The earliest treatment described was open rhinoplasty. Later, some clinicians used nasal endoscopy for the treatment of NDSCs. Nasal endoscopy can enable determination of whether there is an invasion of the nasal cavity and sinuses from the skin incision on the surface of the nasal cavity and mass. It ensures complete removal of the lesion under direct vision, and can clearly evaluate the local tissue defect and abnormal development of the nasal structure after removal of the lesion; thus, it aids in repair of the lesion by the use of corresponding measures during the operation. Skin and cartilage defects can be reconstructed postoperatively by autologous transplantation or transfer of adjacent skin flaps, and the local deformity that develops with age can be reconstructed in the second stage after adulthood. In our study, transnasal endoscopic resection of an intracranial NDSC via an open rhinoplasty approach was used, which achieved a wide surgical exposure with minimal invasiveness and ideal aesthetic results.^[13]^ To target the lesions and intracranial extensions, early craniofacial approach extending to the anterior skull base was adopted; this method has the lowest recurrence rate.^[14–17]^ Open rhinoplasty plus craniotomy is widely used for type III and IV NDSCs. There are some developments in this approach. Various improved surgical methods have been reported, such as double crown craniotomy plus frontal craniotomy, and eyebrow incision and small fenestration craniotomy.^[5]^ The principle of these surgical approaches is to directly expose the intracranial lesions for removal. The lesions can extend from the nose to the intracranial area. According to the size of the lesions, double or single incision may be selected. Furthermore, nasal endoscopy has been used for NDSCs types III and IV. This method is less invasive and reduces the risk of craniotomy. Clinicians can use nasal endoscopy to enter the surgical area from the nasal cavity or via a nasal dorsal incision, reach the skull base, explore the lesion area, resect the lesion, and reconstruct the skull base defect under endoscopy if necessary.^[13,18]^

**Figure 3:**
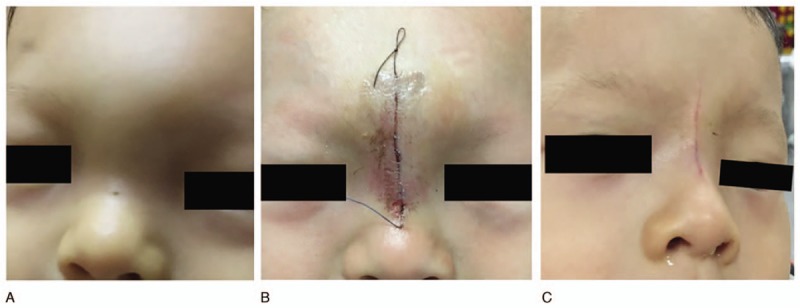
Wound appearance in 1 patient: (A) preoperative appearance of the lesion, (B) wound appearance 3 days postoperatively, and (C) wound appearance 1 month postoperatively.

In our study, type I, type II, and type III lesions were treated by direct resection through a vertical midline incision. In 1 child with postoperative recurrence, 2 vertical incisions were used because the recurrence focus was away from the fistula orifice and not on a vertical line. Another special case underwent a transverse incision at the lower margin of the left eyebrow based on the location of the mass. In a patient with type IV lesion, we performed endoscopic lesion excision and skull base exploration through a vertical midline incision. Complete removal of the lesion was performed in all patients, and there were no serious complications. Parental satisfaction with wound appearance was high (Fig. 3). Our results suggest that most NDSCs can be operated through a vertical midline incision, which is sufficient to fully expose the operative cavity, explore the extent of the lesion, and completely remove it. For patients with intracranial invasion, nasal endoscopy can be used to remove intracranial tumors and repair the skull base. Our choice of surgical pathway was precise, minimally invasive, and had a high success rate; therefore, it can be used as a surgical method for NDSCs in infants and young children.

The only significant complication of early surgery using the craniofacial approach is early infection after surgery, requiring reoperation.^[3]^ However, with advances in surgical techniques, the incidence of complications has been considerably reduced. Pfaff et al^[19]^ reported no complications with the combined cranial and open nasal approach. In our study, 5 patients had low-grade fever postoperatively and 1 patient had high fever after skull base repair. Fever was relieved in all patients after antibiotics treatment, and there were no other complications. This finding may be due to the younger age of the patients (average age 19 months), longer operation time, greater surgical trauma, and reoperations of repairs. None of the 11 children had recurrence or underwent secondary operation. All parents reported that the wound aesthetics were satisfactory. There were very few complications in our patients and the cure rate was 100%. This indicates that the treatment of NDSC in infants and young children can be minimally invasive and highly satisfactory.

According to the preoperative CT findings, 11 patients of NDSCs of the nasal surface type, nasal intraosseous type (including invasion of cartilage and bone), intracranial epidural type, and intracranial dural type in infants and young children were analyzed using the latest classification. All patients were operated using a local vertical incision or combined with minimally invasive nasal endoscopy. There were no serious postoperative complications. The incision was aesthetic and satisfactory, and the prognosis was good. The results of this study provide clinical data for the diagnosis and treatment of NDSCs in infants; however, the number of patients was small, and there were no patients with serious infections or extensive intracranial invasion. Thus, more clinical data are needed to improve the diagnostic and treatment criteria.

## Acknowledgments

The authors thank all members of the Otolaryngology-Head and neck Surgery Department of Shanghai Children's Hospital for their assistance in this study.

## Author contributions

**Conceptualization:** Xiaoyan Li.

**Data curation:** Kun Ni, Linmin Zhao, Xiaojun Liu.

**Methodology:** Kun Ni.

**Project administration:** Xiaoyan Li.

**Resources:** Jiali Wu.

**Software:** Jiali Wu.

**Writing – original draft:** Kun Ni.

## References

[1] Hughes GB Sharpino G Hunt W Management of the congenital midline nasal mass—a review. Head Neck Surg 1980;2:222–33. 7353954 10.1002/hed.2890020308

[2] Littlewood AH Congenital nasal dermoid cysts and fistulas. Plast Reconstr Surg Transplant Bull 1961;27:471–88. 13762755 10.1097/00006534-196105000-00001

[3] Hanikeri M Waterhouse N Kirkpatrick N The management of midline transcranial nasal dermoid sinus cysts. Br J Plast Surg 2005;58:1043–50. 16084501 10.1016/j.bjps.2005.05.021

[4] Meher R Singh I Aggarwal S Nasal dermoid with intracranial extension. J Postgrad Med 2005;51:39–40. 15793337

[5] Hartley BE Eze N Trozzi M Nasal dermoids in children: a proposal for a new classification based on 103 patients at Great Ormond Street Hospital. Int J Pediatr Otorhinolaryngol 2015;79:18–22. 25481331 10.1016/j.ijporl.2014.10.020

[6] Brunner H Harned JW Dermoid cysts of the dorsum of the nose. Arch Otolaryngol 1942;36:86–94.

[7] Sessions RB Nasal dermal sinuses—new concepts and explanations. Laryngoscope 1982;92: 8 pt 2 suppl 29: 1–28. 10.1288/00005537-198208001-00001 7154809

[8] Morgan DW Evans JN Developmental nasal anomalies. J Laryngol Otol 1990;104:394–403. 2370465 10.1017/s0022215100158542

[9] Lin Y Liang Y Liu D A case of nasal dermoid cyst with recessive cranial fissure misdiagnosed as meningocele. Chin Arch Otolaryngol Head Neck Surg 2004;11:194.

[10] Uchida Y Udagawa A Suzuki H The “stepped caudal exposure” technique for excision of nasal dermoids with intracranial extension. J Craniofac Surg 2014;25:648–51. 24621714 10.1097/SCS.0b013e3182a28b1d

[11] Yi B Shi R Wang P Treatment of nasal dorsal midline fistula and congenital dermoid cyst. Chin Arch Otolaryngol Head Neck Surg 2013;20:356–9.

[12] Ortlip T Ambro BT Pereira KD Midline approach to pediatric nasofrontal dermoid cysts. JAMA Otolaryngol Head Neck Surg 2015;141:174–7. 25521829 10.1001/jamaoto.2014.3185

[13] Seidel DU Sesterhenn AM Intracranial nasal dermoid sinus cyst: transnasal endoscopic resection by open rhinoplasty approach, with intraoperative video. J Craniofac Surg 2016;27:2110–2. 28005764 10.1097/SCS.0000000000003107

[14] Yavuzer R Bier U Jackson IT Be careful: it might be a nasal dermoid cyst. Plast Reconstr Surg 1999;103:2082–3. 10359279 10.1097/00006534-199906000-00053

[15] Denoyelle F Ducroz V Roger G Nasal dermoid sinus cysts in children. Laryngoscope 1997;107:795–800. 9185736 10.1097/00005537-199706000-00014

[16] Rohrich RJ Lowe JB Schwartz MR The role of open rhinoplasty in the management of nasal dermoid cysts. Plast Reconstr Surg 1999;104:1459–66. 10513932

[17] Rahbar R Shah P Mulliken JB The presentation and management of nasal dermoid: a 30-year experience. Arch Otolaryngol Head Neck Surg 2003;129:464–71. 12707196 10.1001/archotol.129.4.464

[18] Re M Tarchini P Macrì G Endonasal endoscopic approach for intracranial nasal dermoid sinus cysts in children. Int J Pediatr Otorhinolaryngol 2012;76:1217–22. 22677464 10.1016/j.ijporl.2012.05.004

[19] Pfaff MJ Bickerton S DiLuna M Transcranial nasoethmoidal dermoids: a review and rationale for approach. J Plast Reconstr Aesthet Surg 2013;66:1725–31. 23906597 10.1016/j.bjps.2013.06.039

